# Cross-cultural adaptation of the Internet Gaming Disorder Scale – Short Form (IGDS9-SF) to the Brazilian context

**DOI:** 10.1590/2237-6089-2019-0032

**Published:** 2020-10-08

**Authors:** Mariana F. Donadon, Marcos H. N. Chagas, Thiago D. Apolinário-da-Silva, Erika T. K. Okino, Jaime E. C. Hallak, Êdela A. Nicoletti, Karina Pereira-Lima, Edson A. Degan, Rafael G. Santos, João Paulo Machado-de-Sousa, João L. Q. Simei, Lucas M. Oliveira, Halley M. Pontes, Flávia L. Osório

**Affiliations:** 1 Departamento de Neurociências e Ciências do Comportamento Faculdade de Medicina de Ribeirão Preto Universidade de São Paulo São PauloSP Brazil Departamento de Neurociências e Ciências do Comportamento , Faculdade de Medicina de Ribeirão Preto , Universidade de São Paulo (USP), São Paulo , SP , Brazil .; 2 Departamento de Gerontologia Centro de Ciências Biológicas e da Saúde Universidade Federal de São Carlos São CarlosSP Brazil Departamento de Gerontologia , Centro de Ciências Biológicas e da Saúde , Universidade Federal de São Carlos (UFSCAR), São Carlos , SP , Brazil .; 3 Departamento de Psicologia Faculdade de Filosofia, Ciências e Letras de Ribeirão Preto USP São PauloSP Brazil Departamento de Psicologia , Faculdade de Filosofia, Ciências e Letras de Ribeirão Preto , USP , São Paulo , SP , Brazil .; 4 Centro de Terapia Cognitiva São PauloSP Brazil Centro de Terapia Cognitiva (CTC), Veda, São Paulo , SP , Brazil .; 5 Departamento de Psiquiatria e Psicologia Média Universidade Federal de São Paulo São PauloSP Brazil Departamento de Psiquiatria e Psicologia Média , Universidade Federal de São Paulo (UNIFESP), São Paulo , SP , Brazil .; 6 School of Psychological Sciences University of Tasmania HobartTAS Australia School of Psychological Sciences , University of Tasmania , Hobart TAS , Australia .; 7 The International Cyberpsychology and Addictions Research Laboratory University of Tasmania LauncestonTAS Australia The International Cyberpsychology and Addictions Research Laboratory (iCARL), University of Tasmania , Launceston , TAS , Australia .

**Keywords:** Gaming disorder, internet, scales, cross-cultural adaptation, psychometrics, IGDS9-SF

## Abstract

**Introduction:**

The Internet Gaming Disorder Scale – Short Form (IGDS9-SF) assesses the severity, harmful effects and/or consequences of excessive online and offline gaming. Its conciseness and theoretical foundations on current diagnostic criteria of gaming disorders make it a useful resource for clinical and screening settings.

**Objective:**

To describe the process of cross-cultural adaptation of the IGDS9-SF to the Brazilian context.

**Methods:**

The cross-cultural adaptation involved the steps of independent translation of the instrument, synthesis version, back-translation, pre-test and elaboration of the final version. Content validity assessment was conducted by a multidisciplinary committee of experts and consisted of both a quantitative analysis (calculation of content validity coefficients – CVC) and a qualitative analysis (assessment of the experts’ comments and suggestions). The pre-test sample consisted of 30 gamers with variable sociodemographic characteristics.

**Results:**

The cross-cultural adaptation of the scale followed the proposed protocol, and the CVC was satisfactory (≥ 0.83) for all the structures and equivalences assessed. Most of the suggestions made by the experts were accepted (mainly adjustments and language standardization). The gamers who participated in the pre-test judged the scale easy to understand and did not suggest changes.

**Discussion:**

The Brazilian version of the IGDS9-SF showed adequate content validity and is available for researchers and clinicians, as well as for the investigation of additional psychometric characteristics.

## Introduction

Internet gaming disorder (IGD) can be characterized as a dysfunctional, recurrent and/or problematic pattern of using both online and/or offline video games. ^1 - 3^ More recently, the associated outcomes of dysregulated and disordered gaming have drawn attention from the clinical-scientific community, culminating in the inclusion of this condition as a tentative disorder in the 5th revision of the Diagnostic and Statistical Manual of Mental Disorders (DSM-5). ^3^

According to the DSM-5, IGD denotes persistent use and engagement with online and/or offline video games leading to significant impairment and/or distress (for a minimum of 12 months) as per the endorsement of at least five of the following nine key symptoms: a) concerns about gaming; b) withdrawal symptoms when gaming is interrupted; c) tolerance and/or need to spend increasingly more time gaming; d) failed attempts to control engagement in gaming; e) loss of interest in other activities; f) excessive use of games despite knowledge about associated psychosocial problems; g) lying to family members and/or health professionals in respect to the amount of time spent in gaming; h) use of gaming to escape or relieve negative states; and i) impairment/loss of significant relationships, employment, study, or career opportunities due to gaming. ^3^

The impacts associated with IGD are many, with an emphasis on a) impairments in social, professional, academic, and family activities and relationships ^3 - 5^ ; b) use of gaming as a resource for coping with or procrastinating contact with life difficulties ^2^ ; c) stimulation of violent behaviors when games involve this theme ^6^ ; and d) increasingly hostile behaviors toward the surrounding environment. ^7^

IGD may affect individuals of all ages and its prevalence in adolescents and adults ranges between 10 and 37%, ^8 , 9^ being more predominant among male gamers. ^10 , 11^ The time spent in gaming activities also varies widely, with studies reporting continuous gaming for up to 72 hours a week in disordered gaming cases. ^6^ The comorbid conditions most commonly associated with IGD according to available evidence are major depressive disorder, social anxiety, and attention-deficit hyperactivity disorder. ^12 , 13^

Given the severity, recurrence, and significant impairments associated with IGD, it has become increasingly important to identify and treat affected individuals. However, few instruments are currently available for the screening and assessment of cases. ^14^ Examples include the Problem Video Game Playing (PVP) ^15^ questionnaire, the contents of which do not directly correspond to the current diagnostic criteria of the DSM-5, the Internet Gaming Disorder Scale, ^16^ and the Game Addiction Scale (GAS), ^17 , 18^ which includes a relatively large number of items to be used as a screening instrument.

Conversely, the Internet Gaming Disorder Scale – Short Form (IGDS9-SF), developed by Pontes & Griffiths, ^19^ deserves note for consisting of only nine items derived from the DSM-5 criteria and intended to assess the severity of IGD symptoms, as well as the harmful effects and/or consequences of excessive online and offline gaming.

In the initial development study, the IGDS9-SF was shown to have adequate psychometric properties including criterion validity (moderate correlations with weekly gaming time [r = 0.325] and strong correlations with the Internet Gaming Disorder-20 Test [IGD-20; r = 0.842]) and construct validity (single factor structure). It also presented a moderate reliability indicator (internal consistency, with alpha = 0.87). Furthermore, the IGDS9-SF has already been translated and validated in seven languages: Slovenian, ^20^ Portuguese from Portugal, ^21^ Italian, ^22^ Persian, ^23^ Polish, ^24^ Turkish, and Chinese. In those studies, the psychometric properties of the IGDS9-SF were also found to be adequate (through confirmatory factor analysis), similarly to the original study.

To the best of the authors’ knowledge, there are no instruments available to assess IGD in the Brazilian context. According to Abreu et al., ^2^ studies have assessed IGD using instruments adapted from the diagnostic criteria of substance use disorders due to the lack of a standardized IGD tool in Brazil.

Taken together, the lack of instruments for the assessment of IGD in the Brazilian context, the expressive prevalence and negative impacts of IGD on social functioning, and the increasingly recurrent patterns of gaming misuse highlight the need for resources that allow a wide and brief screening and referral/management of possible cases of IGD. In this context, the IGDS9-SF is a promising tool, which justifies its adaptation and the conduction of psychometric studies in Brazil. Therefore, the aim of this study was to conduct a cross-cultural adaptation of the IGDS9-SF to the Brazilian context and to assess the content validity of the instrument.

## Method

The study was approved by the local ethics committee (process no. 04781318.5.0000.5440). The cross-cultural adaptation was initiated after approval by the original author of the scale (H. M. Pontes), who holds the intellectual property rights of the IGDS9-SF.

The process of translation and adaptation of the IGDS9-SF to Brazilian Portuguese followed the steps proposed by Beaton et al., ^25^ i.e., initial translation, translation synthesis, back-translation, review by an expert committee, and a pilot pre-test. The translation was carried out by three bilingual professionals with expertise in different areas: psychology (KPL), neuroscience (RGS), and languages (English teacher) (EAD), who made independent translations of the instrument. A synthesis version of the three translations (SV1) was then created to resolve discrepancies and to include the terms deemed most adequate for the Brazilian context. This step was performed by two psychologists (FLO, MFD) with previous experience in the fields of psychometrics, psychological assessment, and mental health, who performed the role of judges. The SV1 was then back-translated into English by another Brazilian psychologist (JPMS) with experience in psychopathology. The back-translated version was then examined and approved by the author of the original scale.

Analysis of content validity was performed by an expert committee consisting of two clinical psychologists (ETKO, EAN) and three psychiatrists who also worked as researchers and university professors (JECH, MHNC, TDAS). In each analysis, the experts examined the conceptual, semantic, idiomatic, and cultural equivalence of the instrument as proposed by Beaton et al. ^25^ The analyses were independently performed by each expert and documented in an assessment form developed for the study. The experts were instructed to classify each item according to the following scale: 1 = not equivalent; 2 = with very little equivalence; 3 = somewhat equivalent; 4 = moderately equivalent; and 5 = very equivalent.

The content validity coefficient (CVC) proposed by Hernández-Nieto ^26^ was calculated for each item of the instrument and also as a whole. A cut-off point of ≥ 0.70 was deemed as satisfactory. ^27^ Finally, the judges examined the considerations of the committee, accepting relevant suggestions and devising the final version of the instrument, which was again sent to and approved by the original author.

For the pre-test, a sample of 30 gamers that included both sexes (53.4% male), with age ranging from 18 to 83 years (mean = 35.68, standard deviation = 17.5), and different schooling levels (80% above 9 years of study) was selected by convenience. Participants were instructed to read the instructions, response alternatives, and the content of each item, and then explain what they understood. Subsequently, the gamers were asked if there was any word of difficult understanding and/or if they had any adjustment suggestions.

The process of cross-cultural adaptation of the instrument is described in detail in Table S1, available as online-only supplementary material.

## Results

The mean score obtained for each of the equivalences assessed and the CVC calculated for each of the instrument’s structures are presented in Table 1 .

**Table 1:** Expert agreement indicators for the Brazilian version of the Internet Gaming Disorder Scale – Short Form (IGDS9-SF), detailed by item

Component	Semantic equivalence			Idiomatic equivalence			Cultural equivalence			Conceptual equivalence		
	Mean	(SD)	CVC	Mean	(SD)	CVC	Mean	(SD)	CVC	Mean	(SD)	CVC
Title	4.80	(0.44)	0.96	4.80	(0.44)	0.96	5.00	(0.00)	0.99	5.00	(0.00)	0.99
Instructions	4.80	(0.44)	0.96	4.80	(0.44)	0.96	4.80	(0.44)	0.96	5.00	(0.00)	0.99
Statement 1	5.00	(0.00)	0.99	5.00	(0.00)	0.99	5.00	(0.00)	0.99	5.00	(0.00)	0.99
Statement 2	5.00	(0.00)	0.99	5.00	(0.00)	0.99	5.00	(0.00)	0.99	5.00	(0.00)	0.99
Statement 3	5.00	(0.00)	0.99	5.00	(0.00)	0.99	5.00	(0.00)	0.99	5.00	(0.00)	0.99
Statement 4	5.00	(0.00)	0.99	5.00	(0.00)	0.99	5.00	(0.00)	0.99	5.00	(0.00)	0.99
Statement 5	5.00	(0.00)	0.99	5.00	(0.00)	0.99	5.00	(0.00)	0.99	5.00	(0.00)	0.99
Item 1	4.60	(0.54)	0.92	4.80	(0.44)	0.96	4.80	(0.45)	0.95	5.00	(0.00)	0.99
Item 2	5.00	(0.00)	0.99	5.00	(0.00)	0.99	5.00	(0.00)	0.99	5.00	(0.00)	0.99
Item 3	5.00	(0.00)	0.99	5.00	(0.00)	0.99	4.80	(0.44)	0.96	5.00	(0.00)	0.99
Item 4	5.00	(0.00)	0.99	5.00	(0.00)	0.99	4.80	(0.44)	0.96	5.00	(0.00)	0.99
Item 5	4.20	(0.83)	0.83	4.60	(0.54)	0.92	4.60	(0.89)	0.91	4.80	(0.44)	0.96
Item 6	4.60	(0.89)	0.91	4.60	(0.89)	0.91	4.80	(0.44)	0.96	4.80	(0.44)	0.96
Item 7	4.80	(0.44)	0.96	4.80	(0.44)	0.96	4.80	(0.44)	0.96	4.80	(0.44)	0.96
Item 8	4.60	(0.54)	0.92	4.80	(0.44)	0.96	4.80	(0.44)	0.96	4.40	(0.90)	0.89
Item 9	4.40	(0.89)	0.87	4.60	(0.89)	0.91	4.80	(0.44)	0.96	4.80	(0.44)	0.96
Total	4.80	(1.85)	0.96	4.86	(1.76)	0.97	4.88	(1.17)	0.97	4.91	(0.26)	0.98

CVC = content validity coefficient; SD = standard deviation.

As seen in Table 1 , all components of the IGDS9-SF presented with satisfactory CVC (≥ 0.83), regardless of the type of equivalence assessed. The total CVC of the instrument for the different equivalences was above 0.96.

Experts’ suggestions were minor: in the title, the expression “ *Transtorno de Jogos* ” was changed to “ *Transtorno do Jogo* ” to reflect the definition of the disorder in the DSM-5 and to account for the differences between European and South American Portuguese.

Changes were made in the instructions and on items 4 and 5 to improve the overall cultural equivalence of the instrument. Thus, the word “ *console* ” was replaced by “ *videogame* ,” “ *sistematicamente* ” was replaced by “ *repetidamente* ”, and “ *passatempo* ” was replaced by “ *hobbies* .” Minor adjustments were also made on items 1 and 6 in order to standardize language, adopting “ *atividade de jogo* ” rather than “ *experiência de jogo* ” or “ *hábito de jogar* .” Finally, a minor correction on item 9 of the Portuguese version was made because “ *importante* ” is a qualifier that applies only to the noun “ *relacionamento* ,” and not to the remaining nouns in the sentence.

One final consideration refers to item 5, for which the lowest semantic equivalence CVC was found. When assessing this item, one of the judges rated equivalence as “somewhat equivalent,” arguing that the translation of the word “ *previamente* ” to “ *antigo* ” altered the meaning of the sentence. Although the argument was valid, since the two words are not synonymous according to the Michaelis Portuguese dictionary, ^28^ the suggestion was not incorporated because it was not supported by any of the other experts. In addition, the conceptual and cultural equivalence of the item was quite adequate (CVC = 0.83), which contributed to keep it in its original format.

After these adjustments, the resulting version was sent again to the original author to be assessed, and was then considered the final, official version of the Brazilian IGDS9-SF. The Brazilian version of the IGDS9-SF was named “ *Escala de Transtorno de Jogo pela Internet – Versão Reduzida* ” and can be found in Table S2, available as online-only supplementary material.

In the pre-test stage, the items were easily understood by the sample, and there were no further suggestions of modification in any part of the instrument.

## Discussion

The IGDS9-SF was created to assess symptoms of disordered gaming according to the DSM-5 and has been widely used internationally, with several psychometric validation and adaptation studies to many languages. Until now, the cross-cultural adaptation of the instrument had not been performed in Brazil, a gap that was fulfilled by the present study.

The cross-cultural adaptation of the scale followed all the steps of the method proposed by Beaton et al. ^25^ To accomplish the pre-established steps, a team of translators with different academic backgrounds was formed to increase the chances of finding terms that were most adequate to the wide Brazilian cultural context. The training background of professionals involved in the translation of scales in a process of cross-cultural adaptation is important to preserve the original characteristics of the instrument, making it appropriate for the population with which it is intended to be used. ^28 , 29^

The characteristics of the experts that formed the adaptation committee followed the recommendations made in the literature ^29 , 30^ : all members were bilingual and experts in the fields of psychology and psychiatry, with previous knowledge about the construct under assessment. The use of a quantitative methodology to assess content validity is another strength of this study, as it helps minimizing potential biases due to subjectivity inherent to this type of assessment. This approach helped increase the robustness of the analyses by providing reliable indicators. ^31 - 33^

In addition, the consideration and incorporation of the changes suggested by the experts in the final version of the Brazilian IGDS9-SF were important and support the need for complementary qualitative analyses in these cases. The entire content of the scale was understood by the target audience without difficulties in the pre-test, leading to no further suggestion of change at this stage. It should be emphasized that the pre-test sample was diverse in terms of sex, age and education background, which reinforces the adequacy of the instrument for the general population.

Based on the aforementioned procedures, it is concluded that the Brazilian IGDS9-SF has been adequately adapted to the Brazilian context and had its content validity assessed. Future research should now assess the psychometric properties of the Brazilian IGDS9-SF in terms of its validity and reliability using a robust quantitative methodology, in order to further corroborate the adequacy of the instrument. The IGDS9-SF can be used without the need to request authorization from the authors, whether in clinical or research settings.
