# Supplementary material for: Cross-cultural adaptation of the Internet Gaming Disorder Scale – Short Form (IGDS9-SF) to the Brazilian context
Source: Trends Psychiatry Psychother. 2020 Oct 8;42(3):262–6. doi: 10.1590/2237-6089-2019-0032 (PMC7879067; doi:10.1590/2237-6089-2019-0032)
Supplement: Supplementary file 1 [file 2238-0019-trends-42-03-0262-suppl.pdf]

http://dx.doi.org/10.1590/2237-6089-2019-0032

Table S1 - Process of transcultural adaptation of the IGDS9-SF to Brazilian Portuguese.

	Original English version	Translator 1	Translator 2	Translator 3	Synthesis version (SV1)*	Back-translation	Final version*
**Title**	Internet Gaming Disorder Scale–Short-Form	Escala de Transtorno de Jogos pela Internet – Forma reduzida	Escala de Transtorno de Jogo na Internet– Versão Reduzida	Escala de desordem de jogos na internet- formato curto	Escala de Transtorno de Jogos pela Internet – Versão Reduzida	Internet Gaming Disorder Scale – Short Form	Escala de Transtorno do Jogo pela Internet – Versão Reduzida
**Instructions**	These questions will ask you about your gaming activity during the past year (i.e., last 12 months). By gaming activity we understand any gaming-related activity that has been played either from a computer/laptop or from a gaming console or any other kind of device (e.g., mobile phone, tablet, etc.) both online and/or offline.	As questões abaixo irão lhe fazer perguntas sobre o seu hábito de jogar jogos eletrônicos no último ano (ou seja, nos últimos 12 meses). Por hábito de jogar jogos eletrônicos, nós entendemos qualquer atividade de jogo realizada seja de um computador/notebook, videogame/console, ou ainda de qualquer outro dispositivo eletrônico (por exemplo, celular, tablet, etc.) tanto online quanto offline.	As questões perguntarão sobre suas atividades de jogos no último ano (ou seja, nos últimos 12 meses). Consideramos atividade de jogo como sendo qualquer atividade relacionada a jogos realizada ou em um computador/laptop ou em um console ou em qualquer outro tipo de equipamento (por exemplo, telefone celular, tablet, etc.) online e/ou offline.	Essas perguntas irão lhe perguntar sobre a sua atividade de jogo no ano passado (os últimos 12 meses). Através de atividades de jogos, nos entendemos qualquer atividade relacionada a jogos que tenha sido feita em um computador / laptop (computador portátil) e ou em um console de jogos e ou qualquer outro tipo de dispositivo.(exemplo. Telefone celular, tablet, etc) Ambos conectados e ou desconectados.	Essas questões referem-se a suas atividades de jogo pela internet no último ano (isto é, nos últimos 12 meses). Consideramos atividade de jogo como sendo qualquer atividade relacionada a jogos realizada ou em um computador/laptop ou em um console ou em qualquer outro tipo de equipamento (por exemplo, telefone celular, tablet, etc.) online e/ou offline.	These questions refer to your Internet gaming activities in the past year (that is, in the last 12 months). We regard gaming as any activity related to games performed either on a computer/laptop or console or any other type of device (for example, cell phone, tablet etc.), online and/or offline.	Essas questões referem-se a sua atividade de jogo no último ano (isto é, nos últimos 12 meses). Consideramos atividade de jogo como sendo qualquer atividade relacionada a jogos realizada ou em um computador/laptop ou em um videogame ou em qualquer outro tipo de equipamento (por exemplo, telefone celular, tablet, etc.) online e/ou offline

**Statement**	Never, rarely, sometimes, often, very often	Nunca, raramente, algumas vezes, frequentemente, muito frequentemente	Nunca, raramente, algumas vezes, frequentemente, muito frequentemente	Nunca, raramente, algumas vezes, frequentemente, muito frequentemente	Nunca, raramente, algumas vezes, frequentemente, muito frequentemente	Nunca, raramente, algumas vezes, frequentemente, muito frequentemente	Nunca, raramente, algumas vezes, frequentemente, muito frequentemente
**Item 1**	1. Do you feel preoccupied with your gaming behavior? (Some examples: Do you think about previous gaming activity or anticipate the next gaming session? Do you think gaming has become the dominant activity in your daily life?)	1. Você se preocupa com o seu hábito de jogar jogos eletrônicos? (Por exemplo: Você pensa sobre uma experiência de jogo passada ou planeja a próxima vez que irá jogar? Você acha que jogar jogos eletrônicos se tornou a atividade dominante do seu cotidiano?	1. Você se sente preocupado como seu comportamento de jogo? (Alguns exemplos: Você pensa sobre atividades de jogos prévias ou antecipa a próxima sessão de jogo? Você acha que o jogo se tornou a atividade principal do seu dia a dia?)	1.Voce se sente preocupado com seu comportamento de jogo? (Alguns exemplos: você penso sobre as atividades anteriores de jogos ou antecipa a próxima sessão de jogos? Você acredita que jogar se tornou uma atividade dominante em sua vida diária?	1. Você se sente preocupado com o seu comportamento de jogo? (Por exemplo: Você pensa sobre uma <u>experiência</u> de jogo passada ou antecipa a próxima vez que irá jogar? Você acha que o jogo se tornou a atividade principal do seu dia a dia?)	1. Do you have concerns about your gaming behavior? (For example: Do you think about a past gaming experience or anticipate the next time you will play? Do you feel that gaming has become the main activity of your daily life?)	1. Você se sente preocupado com o seu comportamento de jogo? (Por exemplo: Você pensa sobre uma <u>atividade</u> de jogo passada ou antecipa a próxima vez que irá jogar? Você acha que o jogo se tornou a atividade principal do seu dia a dia?)
**Item 2**	2. Do you feel more irritability, anxiety or even sadness when you try to either reduce or stop your gaming activity?	2. Você sente mais irritabilidade, ansiedade ou até mesmo tristeza quando você tenta reduzir ou encerrar o seu hábito de jogar jogos eletrônicos?	2. Você sente mais irritabilidade, ansiedade ou até mesmo tristeza quando tenta reduzir ou parar sua atividade de jogo?	2.Voce sente mais irritabilidade, ansiedade e ou ate mesmo tristeza quando você tenta tanto parar e ou ate mesmo reduzir suas atividades de jogos?	2. Você sente mais irritabilidade, ansiedade ou até mesmo tristeza quando tenta reduzir ou parar sua atividade de jogo?	2. Do you feel more irritable, anxious or even sad when you try to reduce or quit gaming?	2. Você sente mais irritabilidade, ansiedade ou até mesmo tristeza quando tenta reduzir ou parar sua atividade de jogo?
**Item 3**	3. Do you feel the need to spend increasing amount of time engaged gaming in order to achieve satisfaction or pleasure?	3. Você sente que precisa passar cada vez mais tempo jogando jogos eletrônicos para obter satisfação ou prazer?	3. Você sente a necessidade de passar cada vez mais tempo envolvido em jogos para conseguir satisfação ou prazer?	3.Voce sente a necessidade de gastar uma quantidade crescente de tempo envolvido no jogo, para que assim alcance satisfação e ou prazer?	3. Você sente a necessidade de passar cada vez mais tempo envolvido em jogos para conseguir satisfação ou prazer?	3. Do you feel the need to spend increasingly more time involved in games in order to get satisfaction or pleasure?	3. Você sente a necessidade de passar cada vez mais tempo envolvido em jogos para conseguir satisfação ou prazer?

**Item 4**	4. Do you systematically fail when trying to control or cease your gaming activity?	4. Você falha sistematicamente quando tenta controlar ou parar com o seu hábito de jogar jogos eletrônicos?	4. Você falha sistematicamente quando tenta controlar ou cessar sua atividade de jogo?	4.Voce falha sistematicamente ao tentar se controlar e ou cessar suas atividades de jogos?	4. Você falha sistematicamente quando tenta controlar ou cessar sua atividade de jogo?	4. Do you systematically fail when trying to control or quit gaming?	4. Você falha repetidamente quando tenta controlar ou cessar sua atividade de jogo?
**Item 5**	5. Have you lost interests in previous hobbies and other entertainment activities as a result of your engagement with the game?	5. Você perdeu o interesse em antigos *hobbies* (passatempos) e outras atividades que fazia para se divertir como consequência do seu envolvimento com o jogo?	5. Você perdeu interesse em hobbies anteriores e em outras atividades de entretenimento como resultado de seu envolvimento com jogos?	5.Voce já perdeu interesse em hobbies antigos e outras atividades de entretenimento como resultado do engajamento com o jogo?	5. Você perdeu o interesse em antigos passatempos e outras atividades que fazia para se divertir como resultado do seu envolvimento com o jogo?	5. Have you lost interest in old hobbies and other activities you did to have fun as a result of your involvement with gaming?	5. Você perdeu o interesse em antigos hobbies e outras atividades que fazia para se divertir como resultado do seu envolvimento com jogo?
**Item 6**	6. Have you continued your gaming activity despite knowing it was causing problems between you and other people?	6. Você manteve o seu hábito de jogar jogos eletrônicos apesar de saber que isso estava causando problemas entre você e as outras pessoas?	6. Você continuou sua atividade de jogo mesmo sabendo que isso estava causando problemas entre você e outras pessoas?	6.Voce já continuou sua rotina de jogos mesmo sabendo que isso estava causando problemas entre você e outras pessoas?	6. Você manteve o seu hábito de jogar apesar de saber que isso estava causando problemas entre você e as outras pessoas?	6. Have you kept your gaming habits despite knowing that this was causing problems between you and others?	6. Você manteve a sua atividade de jogo apesar de saber que isso estava causando problemas entre você e as outras pessoas?
**Item 7**	7. Have you deceived any of your family members, therapists or others because the amount of your gaming activity?	7. Você enganou algum membro da sua família, terapeutas ou outras pessoas devido a quantidade que você joga jogos eletrônicos?	7. Você enganou algum de seus familiares, terapeutas ou outras pessoas pela quantidade de sua atividade de jogo?	7.Voce já enganou algum membro de sua família, terapeutas e ate mesmo outros devido a quantidade de sua atividade de jogo?	7. Você enganou algum familiar, terapeuta ou outras pessoas em relação ao tanto que você joga?	7. Have you fooled any relatives, therapists or other people due to the amount you play?	7. Você enganou algum familiar, terapeuta ou outras pessoas devido ao tanto que você joga?

**Item 8**	8. Do you play in order to temporarily escape or relieve a negative mood (e.g., helplessness, guilt, anxiety)?	8. Você joga para aliviar ou escapar temporariamente de uma emoção negativa (por exemplo, impotência, culpa, ansiedade)?	8. Você joga para escapar temporariamente ou aliviar um sentimento negativo (por exemplo, desesperança, culpa, ansiedade)?	8.Voce joga para escapar temporariamente ou aliviar seu humor negativo (exemplo; desamparo, culpa, ansiedade?	8. Você joga para aliviar ou escapar temporariamente de uma emoção negativa (por exemplo, desamparo, culpa, ansiedade)?	8. Do you play in order to relieve or temporarily evade a negative emotion (for example, hopelessness, guilt, anxiety)?	8. Você joga para escapar ou aliviar temporariamente uma emoção negativa (por exemplo, desamparo, culpa, ansiedade)?
**Item 9**	9. Have you jeopardized or lost an important relationship, job or an educational or career opportunity because of your gaming activity?	9. Você prejudicou ou perdeu um relacionamento importante, emprego ou uma oportunidade educacional ou de carreira devido ao seu hábito de jogar jogos eletrônicos?	9. Você prejudicou ou perdeu uma relação importante, um emprego ou uma oportunidade educativa ou de carreira devido a sua atividade de jogo?	9.Voce já comprometeu e ou já perdeu relações importantes, trabalho, ou educacional ou oportunidade de carreira devido a sua atividade de jogos?	9. Você prejudicou ou perdeu um relacionamento, um emprego, uma oportunidade educacional ou de carreira importante devido a sua atividade de jogo?	9. Have you harmed or lost an important relationship, job, or educational or career opportunity because of your gaming?	9. Você prejudicou ou perdeu um relacionamento importante, emprego ou uma oportunidade educacional ou de carreira devido a sua atividade de jogo?

* Underlined type: changes made.

Table S2 - *Escala de Transtorno do Jogo pela Internet – Versão Reduzida* (IGDS9-SF) (Pontes & Griffiths, 2015)

Instruções: Essas questões referem-se a sua atividade de jogo no último ano (isto é, nos últimos 12 meses). Consideramos atividade de jogo como sendo qualquer atividade relacionada a jogos realizada ou em um computador/laptop ou em um videogame ou em qualquer outro tipo de equipamento (por exemplo, telefone celular, tablet, etc.) online e/ou offline

	**Nunca**	**Raramente**	**Algumas vezes**	**Frequente mente**	**Muito frequentement e**
1. Você se sente preocupado com o seu comportamento de jogo? (Por exemplo: Você pensa sobre uma atividade de jogo passada ou antecipa a próxima vez que irá jogar? Você acha que o jogo se tornou a atividade principal do seu dia a dia?)	○	○	○	○	○
2. Você sente mais irritabilidade, ansiedade ou até mesmo tristeza quando tenta reduzir ou parar sua atividade de jogo?	○	○	○	○	○
3. Você sente a necessidade de passar cada vez mais tempo envolvido em jogos para conseguir satisfação ou prazer?	○	○	○	○	○
4. Você falha repetidamente quando tenta controlar ou cessar sua atividade de jogo?	○	○	○	○	○
5. Você perdeu o interesse em antigos *hobbies* e outras atividades que fazia para se divertir como resultado do seu envolvimento com jogo?	○	○	○	○	○
6. Você manteve a sua atividade de jogo apesar de saber que isso estava causando problemas entre você e as outras pessoas?	○	○	○	○	○
7. Você enganou algum familiar, terapeuta ou outras pessoas devido ao tanto que você joga?	○	○	○	○	○
8. Você joga para escapar ou aliviar temporariamente uma emoção negativa (por exemplo, desamparo, culpa, ansiedade)?	○	○	○	○	○
9. Você prejudicou ou perdeu um relacionamento importante, emprego ou uma oportunidade educacional ou de carreira devido a sua atividade de jogo?	○	○	○	○	○

Informações sobre o Escore:

A pontuação total pode ser obtida a partir da soma das respostas dadas aos nove itens da IGDS9-SF, e pode variar de um mínimo de 9 a um máximo de 45 pontos, com pontuações mais altas sendo indicativas de um nível mais elevado do Transtorno do Jogo pela Internet. Para diferenciar jogadores com e sem o transtorno, os pesquisadores devem checar se os participantes assinalaram pelo menos cinco dos nove critérios com a resposta *"5: muito frequentemente"*, o que ratifica o critério diagnóstico.

Referência:

Pontes, H. M., & Griffiths, M. D. (2015). Measuring DSM-5 Internet Gaming Disorder: Development and validation of a short psychometric scale. *Computers in Human Behavior, 45*, 137-143. doi:10.1016/j.chb.2014.12.006

Tradução para o português do Brasil:

Osório FL; Donadon MF; Chagas MHN; Apolinário TD; Okino ETK; Hallak JEC, Nicholleti, E; Pereira-Lima K; Degan EA; Santos RG; Sousa JPM. 2018. Com autorização
